# Genomic Observations of a Rare/Pathogenic *SMAD3* Variant in Loeys–Dietz Syndrome 3 Confirmed by Protein Informatics and Structural Investigations

**DOI:** 10.3390/medicina55050137

**Published:** 2019-05-15

**Authors:** John E. Richter, Ayesha Samreen, Charitha Vadlamudi, Haytham Helmi, Ahmed N. Mohammad, Klaas Wierenga, Stephanie Hines, Paldeep S. Atwal, Thomas R. Caulfield

**Affiliations:** 1 Department of Clinical Genomics, Mayo Clinic, Jacksonville, FL 32224, USA; jrichter133@ufl.edu (J.E.R.J.); ahmed.nabil.mohammad@gmail.com (A.N.M.); Wierenga.Klaas@mayo.edu (K.W.); DrA@atwalclinic.com (P.S.A.); 2 Department of Endocrinology, Mayo Clinic, Jacksonville, FL 32224, USA; Samreen.Ayesha@mayo.edu; 3 Department of Pulmonology and Critical Care, Mayo Clinic, Jacksonville, FL 32224, USA; Vadlamudi.Charitha@mayo.edu (C.V.); Helmi.Haytham@mayo.edu (H.H.); 4 Department of Internal Medicine, Mayo Clinic, Jacksonville, FL 32224, USA; Hines.Stephanie@mayo.edu; 5 Department of Neuroscience, Mayo Clinic, Jacksonville, FL 32224, USA; 6 Department of Neurosurgery, Mayo Clinic, Jacksonville, FL 32224, USA; 7 Department of Cancer Biology, Mayo Clinic, Jacksonville, FL 32224, USA; 8 Department of Health Sciences Research, Mayo Clinic, Jacksonville, FL 32224, USA

**Keywords:** SMA- and MAD-related protein 3 (SMAD3), Loeys–Dietz syndrome 3 (LDS3), protein informatics, molecular genomics, pathogenicity, case report

## Abstract

*Background and objectives:* Loeys–Dietz syndrome 3, also known as aneurysms-–osteoarthritis syndrome, is an autosomal dominant genetic connective tissue disease caused by pathogenic variants in *SMAD3*, a transcription factor involved in TGF-β signaling. This disorder is characterized by early-onset osteoarthritis and arterial aneurysms. Common features include scoliosis, uvula abnormalities, striae, and velvety skin. *Materials and Methods*: The pathogenicity of a variant of uncertain significance in the *SMAD3* gene was evaluated (variant c.220C > T) through personalized protein informatics and molecular studies. *Results:* The case of a 44-year-old male, who was originally presumed to have Marfan syndrome, is presented. An expanded gene panel determined the probable cause to be a variant in *SMAD3*, c.220C > T (p.R74W). His case was complicated by a history of stroke, but his phenotype was otherwise characteristic for Loeys–Dietz syndrome 3. *Conclusion:* This case emphasizes the importance of comprehensive genetic testing to evaluate patients for connective tissue disorders, as well as the potential benefit of utilizing a protein informatics platform for the assessment of variant pathogenicity.

## 1. Introduction

SMAD family member 3 (*SMAD3*), also known as mothers against decapentaplegic homolog 3, is a protein encoded at locus 15q22.33 that is implicated in transforming growth factor-beta (TGF-β) signaling (OMIM*603109). TGF-β is responsible for regulating certain cellular functions, including apoptosis, angiogenesis, and cell growth. This is achieved through various signaling pathways, and the SMAD signaling pathway is one of many. *SMAD3* is a receptor-activated SMAD, meaning that it is phosphorylated by the transmembrane proteins binding to TGF-β. Once activated, *SMAD3* binds to *SMAD4*, which chaperones the protein to the nucleus. There, *SMAD3* is able to regulate the transcription of TGF-β target genes [1]. Wild-type *SMAD3* function is essential for TGF-β to properly express these genes and perform its wide range of signaling responsibilities. The consequences of improper TGF-β signaling are reflected in Loeys–Dietz syndrome 3 (LDS3) (OMIM#613795), a multisystem connective tissue disorder caused by pathogenic variants of *SMAD3* [2]. The Human Gene Mutation Database (HGMD) currently lists 69 unique variants within this gene, most of which are missense/nonsense variants.

The prevalence of Loeys–Dietz syndrome is unknown. First described in 2005, it is a recently discovered connective tissue disorder with multisystem involvement (PMID 15731757). Also known as aneurysms–osteoarthritis syndrome, LDS3 most notably causes premature osteoarthritis and arterial aneurysms. Osteoarthritis tends to be the first sign of LDS3. This symptom distinguishes LDS3 from the other forms of Loeys–Dietz syndrome, which are not typically associated with joint degeneration [3]. Tortuosity often accompanies arterial aneurysms in LDS3. These aneurysms most commonly affect the aorta, but other arteries may also be involved [2]. Sudden arterial dissection is the cause of death for some patients. Craniofacial deformities, including uvula abnormalities and hypertelorism, are sometimes present. Skeletal abnormalities such as scoliosis are common in LDS3, as are cutaneous conditions including striae and velvety skin [3]. A comprehensive table is provided to summarize LDS3-related diseases (Table 1). The five types of Loeys–Dietz syndrome are briefly described in a second table (Table 2).

This report discusses a male presenting with a LDS3 phenotype and a rare *SMAD3* variant, denoted c.220C > T (p.R74W). Molecular modeling was utilized to evaluate the pathogenicity of this variant. Additionally, we provide support for the use of large gene-panel testing to ensure accurate diagnosis and properly inform medical management.

### Clinical Description

The proband was a 44-year-old male who was previously evaluated for Marfan syndrome. His presenting features were aortic aneurysm and tall stature (6’3”). He reported that his aneurysm was first measured around 17 years ago at 4.2 cm in diameter. Surgical intervention was not required until age 35, at which point the aneurysm had increased to 6.0 cm in diameter. The proband underwent an ascending aortic aneurysm repair with a mechanical aortic valve. Afterward, his aortic root measured 3.3 cm in diameter. However, he experienced a stroke complicated by transient ischemic attacks the following year. The stroke was potentially associated with the proband’s patent foramen ovale, which was discovered and closed in the aftermath of the stroke. This series of events prompted the proband to seek a medical genetics evaluation 7 years ago. A physical exam revealed striae on the groin and anterior to the axillae, corrected tooth crowding, and mild scoliosis. The absence of lens abnormalities challenged the diagnosis of Marfan syndrome, but sequencing of *FBN1*, *TGFBR1*, and *TGFBR2* was performed nonetheless. No pathogenic variant was detected, though one intronic variant of uncertain significance was reported in *TGFBR2*. The absence of an apparent genetic defect caused the proband to discontinue visits to the clinic. Recently, the proband had an exercise electrocardiogram (ECG) during a routine work evaluation that showed rapid sinus rhythm. This caused him to revisit his condition and seek evaluation in our clinic.

In the time since his first genetic testing, the proband developed a significant degree of osteoarthritis. He underwent left hip replacement and knee surgery to relieve associated pains. Double vision and occasional spots in his vision were also reported. The proband was evaluated on-site by an ophthalmologist, who discovered no ocular manifestations of a connective tissue disease. Family history revealed bifid uvula in the proband’s son and suspected ectopia lentis in his mother. Based on his reported personal and family history, a connective tissue disorder was still suspected. Consequently, a large panel of connective tissue-related genes was ordered (Table 1). A previously missed variant in *SMAD3* was identified. The *SMAD3* variant, c.220C > T (p.R74W), was classified as a variant of uncertain significance by the genetic testing laboratory. Another variant of uncertain significance was reported in *FBN2*, c.8376 C > G (p.I2792M), but this variant was not thought to be clinically relevant. The presence of the suspicious *SMAD3* variant, aortic aneurysm, and osteoarthritis suggested that LDS3 was the causal diagnosis. The pathogenicity of the p.R74W variant in *SMAD3* was further supported by the results of molecular modeling.

## 2. Materials and Methods

### 2.1. Protein Informatics and Molecular Modeling

Our methodology has been documented previously in the literature [4,5,6,7,8,9]. The sequence of the human protein SMAD3, a protein encoded by the *SMAD3* gene, was taken from the NCBI Reference Accession Sequence: NM_005902: version NM_005902.3, and was used for computer-assisted modeling. Monte Carlo simulations were performed on the mutant to allow local regional changes for full-length 425 amino acids and when the p.R74W variant was introduced. The protein forms a trimeric (homo-trimeric) complex in the structural modeling (homomeric), as is shown to be the case from the X-ray structural data set. SMAD3 is known to form partner complexes with other proteins (*SMAD4*, JUN, or FOS) to form complexes that initiate transcription. Mutations disrupting the interaction of the trimeric complex may play a role in the dysfunction of this protein in genetically predisposed individuals. The Protein DataBank (PDB) codes used for building the composite hybrid model for optimal structure include the following: 1MHD, 1MJS, 1MK2, 1OZJ, 1U7F, 2LAJ, 2LB2, 5OD6, 5ODG, and 5XOC.

The X-ray refinement using a Monte Carlo (MC) algorithm to search for optimal conformations of the structure was built using our YASARA SSP/PSSM Method [10,11,12,13,14,15] to obtain the initial structure. MC searches were then completed for lower saddle point energy and equilibrated with brief molecular dynamics simulations to allow relaxation of the structure in an aqueous environment. The structure was relaxed to the YASARA/Amber force field using knowledge-based potentials within YASARA. The side chains and rotamers were adjusted with knowledge-based potentials, simulated annealing with an explicit solvent, and performed small equilibration simulations using YASARA’s refinement protocol [16]. The entire full-length structure was modeled, filling in any gaps or unresolved portions from the X-ray.

The refinement of the finalized models was completed using either Schrodinger’s LC-MOD Monte Carlo-based module or NAMD2 protocols. These refinements started with YASARA-generated initial refinement and mutant R74W [10,11,12,14]. The superposition and subsequent refinement of the overlapping regions yields a complete model for *SMAD3*. The final structures were subjected to energy optimization with Polak–Ribiére conjugate gradient (PR-CG) with an R-dependent dielectric.

Atom consistency was checked for all dimeric complexes of 425 amino acids as a trimeric complex (20,019 atoms) of the full-length wild-type model and number for the W74 variant, verifying the correctness of chain name, dihedrals, angles, torsions, non-bonds, electrostatics, atom-typing, and parameters. Each model was exported to the following formats: Maestro (MAE) and YASARA (PDB). Model manipulation was performed with Maestro (Macromodel, version 9.8, Schrodinger, LLC, New York, NY, USA), or Visual Molecular Dynamics (VMD) [17].

Monte Carlo dynamics searching (LCMOD-MC) was completed on each model for conformational sampling, using methods previously described in the literature [18,19,20,21]. Briefly, each *SMAD3* wild-type or variant system was minimized with relaxed restraints using either Steepest Descent or Conjugate Gradient PR, then allowed to undergo the MC search criteria, as shown in the literature [18,19,20,21]. The primary purpose of Monte Carlo, in this scenario, is examining any conformational variability that may occur with different mutation in the region near to the mutation and possible effect on trimeric formation or partner protein binding with *SMAD3*.

### 2.2. Consent for Publication and Informed Consent

All procedures followed were in accordance with the ethical standards of the responsible committee on human experimentation (institutional and national) and with the Helsinki Declaration of 1975, as revised in 2000 (5), (project identification code: 7972201). Informed written consent was obtained from the patient included in the study.

## 3. Results

### 3.1. Molecular Modeling and Thermodynamic Measurements

**Figure 1:**
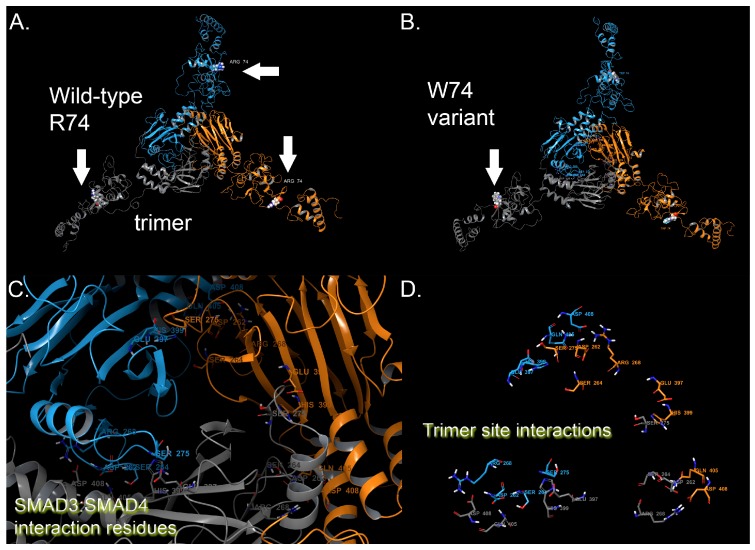
SMAD3 molecular model for the full-length human sequence consisting of 425 amino acids and variant p.R74W. (**A**) Full-length model for the entire SMAD3 structure that shows trimer interaction with position 74 indicated by arrows. Each monomer of the trimer is indicated by a different color of ribbon (blue, orange, gray). (**B**) p.R74W variant for SMAD3. W74 is indicated by arrows. (**C**) Enlarged (zoomed into) the interaction region for SMAD3, where trimers are visible and also where SMAD4 can form heterodimers. Homodimerization and interaction residues are shown, colored by a SMAD3 monomer (blue, orange, gray). (**D**) "Ribbonless" view of the trimer interaction residues is shown. All protein residues shown in licorice rendering and using standard element coloring (O-red, N-blue, H-white, S-yellow), where carbons are shown in orange, blue or gray to indicate the monomer derived for trimer.

Examinations of the trimer reveal an interaction zone that consists of residues, shown in Figure 1C–D, that forms a pore-like circular interface (two sheets of anti-parallel beta sheets layered onto two alpha helices) (Figure 1C–D). These residues are known to be critical for the formation of *SMAD4* heterodimerization contacts, which is crucial for the function in transcription regulation (Figure 1D).

**Figure 2:**
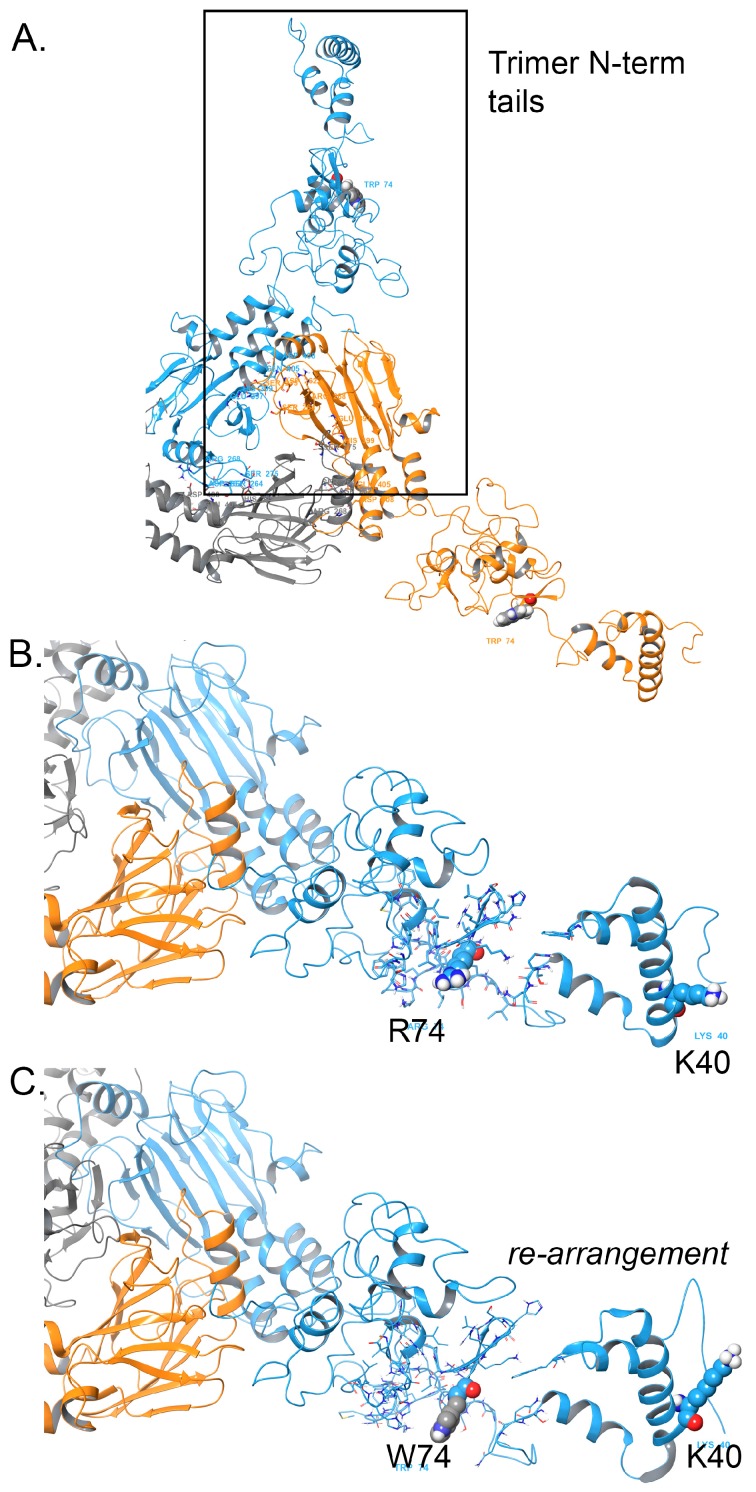
Rearrangement of the SMAD3 region around p.R74W. (**A**) Full-length model for the entire SMAD3 structure that shows trimer interaction with position 74 boxed in for emphasis. (**B**) Wild-type for SMAD3 showing the native conformation for the N-terminus with R74 and K40 for reference. (**C**) W74 variant for SMAD3 is shown. Here, the rearrangement causes conformational changes including K40 repositioning. Colors, rendering and style are the same as Figure 1.

The N-terminus half maintains a different set of residues around R74 (wild-type) that are critical for complex formation (Figure 2), namely residues: Trp18, Lys40, Thr57, Gln58, Asn59, Ile67, Pro68, Arg69, Ser70, Leu71, Asp72, Gly73, Arg74, Leu75, Gln76, Val77, Ser78, His79, Arg80, Lys81, Gly82, Leu83, Pro84, His85, Val86, Ile87, Tyr88, Cys89, Arg90, Leu91, Asp96, Leu97, His98, Ser99, His100, His101, Leu103, Phe111, Ala112, Phe113, Asn114, Met115, Lys116, Lys117, Asp118, Glu119, Val120, and Val122. The introduction of the W74 residue demonstrates disruption on the structure around this region, which is visually evident (Figure 2B,C) [18,19,20,22,23,24,25]. Additionally, we measured the root-mean-square-deviation (RMSD) for the residues mentioned above between the wild-type and variant structure, which revealed a large structural deviation due to the mutation. The RMSD for the 6 Å cutoff around the residues given above (centered on positions 74 and 40) resulted in 16.19 Å deviation for all residues, 13.09 Å for the backbone residues, and 8.08 Å for the C-alpha residues only. By any metric of RMSD, those are significant changes indicative of a different conformational state that has the potential to alter function.

### 3.2. Energetics Assessment

For wild type versus the variant p.R74W, we found that the stability of the object from energetic calculations for ΔG per amino acid is lower for the R74W variant, such that p.R74W was 0.0979 kcal/aa × mol × Å^2^ greater than wild-type, respectively [18,19,20,22,23,24,25]. We examined wild-type object stability, obtaining 673.1 kcal/mol × Å^2^, which is normal for a protein of 425 aa size (Figure 2A). This object stability did not indicate any changes in structure that were deleterious to function from immediate inspection, which the mutation Fold-X algorithm can provide. However, the W74 mutation in the N-terminus region is positive, thus disruptive, and the MC analysis showed some significant rearrangement for the N-terminus around residues 40 through 90, which are demonstrably more prone to conformational variability and play an important role in proper complex formation (Figure 2B,C). However, the protein with p.R74W results in a change of protein function that could come as a consequence of the alteration of the region of residues near to K40 and also the general interaction around the region of W74. The molecular model for the full structure and its truncated form are given (Figure 1A,B and Figure 2A) using our state-of-the-art methods, which have been established [18,19,20,21,22,25,26,27,28,29,30,31,32].

## 4. Discussion

The proband’s aortic aneurysm and osteoarthritis are characteristic of LDS3. His physical abnormalities, striae and scoliosis, are also commonly reported [3]. However, most LDS3 patients experience osteoarthritis before having an aneurysm. Our proband did not develop osteoarthritis for over ten years after his aneurysm was first discovered. Additionally, his patent foramen ovale is unique among other reported LDS3 patients, though other congenital heart defects such as patent ductus arteriosus and atrial septal defect have been recorded [2,3,33]. Regardless, we cannot rule out the possibility that this defect was idiopathic. Around 25% of adults who retain a patent foramen ovale from birth do not possess an underlying disorder [34]. Diplopia was the final symptom that the proband reported. Considering his history of stroke, it seems unlikely that this is a newly found LDS3 symptom [35]. Altogether, the proband presents a largely typical case of LDS3 that is complicated by the symptoms of a past stroke.

Recent research in mouse models provides us with clues to unravel the mechanisms behind aortic aneurysms in LDS3 patients. A study by Tan et al. linked aortic inflammation and high macrophage numbers to weakness of the aortic wall. Inducible nitric oxide synthase (iNOS) activity in these macrophages is increased. In turn, the nitric oxide product of iNOS activates matrix metalloproteinases 2 and 9. These proteinases break down elastic lamella and collagen fibers in nearby extracellular matrices, leading to aortic wall weakness and susceptibility to rupturing or dissection [36]. Another study using *SMAD3*-deficient mice suggested that wild-type *SMAD3* is necessary to prevent macrophage intrusion into arterial walls. In this study, matrix metalloproteinase mediated the decay of elastic fibers, which was observed only in *SMAD3*-deficient individuals. This further supported the previous findings of Tan et al. The researchers also found an increased expression of nuclear factor kappa-B, *SMAD2/SMAD4* and TGF-β1, and ERK1/2 [37]. These studies suggest that preventing aortic rupture and death of *SMAD3*-deficient patients may be possible through inhibition of overexpressed protein products or signaling pathways. Particularly, iNOS antagonists are proposed as a solution to some arterial wall weakness, as inhibiting this enzyme would reduce matrix metalloproteinase activation and elastic fiber degradation [36]. To date, a drug with this function has yet to be approved. The current option for treating LDS3-related aneurysms is surgery, though this had limited success for our proband.

The arginine residue in position 74 (Arg74) is located in the MH1 domain of the *SMAD3* protein. The MH1 domain is a conserved N-terminal domain and is required for DNA binding. Arg74 is a key DNA binding residue as it directly interacts with the guanosine in the GTCT Smad binding element. Qing et al. (2000) found that substituting Arg74 for Aspartic acid (Asp) can result in the failure of *SMAD3* to cooperate with c-jun leading to a loss of transcriptional activity [38].

The R74W variant is a non-conservative amino acid substitution, which is likely to impact secondary protein structure. This substitution occurs at a position that is conserved across species, and in silico analysis predicts this variant to be probably damaging to the protein structure/function. Polyphen-2 bioinformatics tool predicted the R74W variant to be probably damaging with a score of 1.000.

The *SMAD3* trimer has a native conformation that is stable and suitable for binding with key partners, such as *SMAD4*, required for transcription initiation, which is likely affected through the mutation at position 74, which is proximal to K40 (Figure 2C). K40 is a known residue of importance for complex formation. We observed disruption in the local region around the K40 and through residues Val120, which could in turn disrupt the important regions needed for partner binding (Figure 1C).

This proband first presented with Marfan-like features, including aortic aneurysm, tall stature, tooth crowding, striae, and scoliosis. This phenotype was indicative of a general connective tissue disorder. Despite this, the proband was initially tested for just three genes, after which no further sequencing was pursued. These tests only ruled out Marfan syndrome and Loeys–Dietz syndrome 1&2 from his differential diagnosis. Consequentially, it took the proband seven years from the time of his first clinical investigation to learn about the disorder responsible for his condition. Had his final diagnosis been treatable, this would have greatly hindered the management of his symptoms. Nevertheless, closely managing blood pressure is still helpful for mitigating the symptoms of LDS3. An earlier diagnosis may have allowed the proband to take necessary action to improve his condition and possible long-term outcomes (36).

## 5. Conclusions

In conclusion, we report a proband with a MH1 domain, pathogenic variant in *SMAD3* and compare his phenotype to other patients with LDS3. The proband’s clinical presentation fits well with LDS3, though his condition was complicated by a concurrent stroke. The proband’s son has a 50% chance to carry the same *SMAD3* variant, but we suspect the probability is higher due to his bifid uvula. Close observation of the proband’s son will be necessary to detect warning signs of LDS3 in the future. The c.220C > T (p.R74W) variant is suspected to be pathogenic based on the results of personalized molecular modeling. Our proband’s *SMAD3* variant was only detected following a more comprehensive connective tissue disease gene panel. The delay of diagnosis during his diagnostic odyssey was likely due to multiple factors, but it exemplifies the importance of comprehensive disease gene analysis in clinical practice when appropriate.

## Figures and Tables

**Table 1:** Genes evaluated in heritable disorders of connective tissue (HDCT) sequencing and deletion/duplication panel.

Gene	Protein	Inheritance	Disease Association(s)
*ACTA2*	Actin, alpha-2, smooth muscle, aorta	AD	fTAAD
*ADAMTS2*	Adam metallopeptidase with thrombospondin type 1 motif 2	AR	dEDS
*ALDH18A1*	Aldehyde dehydrogenase 18 family member a1	AD AR	Cutis laxa
*ATP6V0A2*	Atpase h+ transporting v0 subunit a2	AR	Cutis laxa
*ATP7A*	Atpase copper transporting alpha	XL	Menkes, Obesity hypoventilation syndrome (OHS)
*B3GALT6*	Beta-1,3-galactosyltransferase 6	AR	spEDS
*B4GALT7*	Beta-1,4-galactosyltransferase 7	AR	spEDS
*CBS*	Cystathionine beta-synthase	AR	Homocystinuria
*CHST14*	Carbohydrate (dermatan 4) sulfotransferase 14	AR	mcEDS
*COL11A1*	Collagen type xi alpha 1	AD	Fibrochondrogenesis Stickler syndrome
*COL11A2*	Collagen type xi alpha 2	AD	Fibrochondrogenesis Stickler syndrome, non-ocular
*COL1A1*	Collagen type i alpha 1	AD	aEDS cEDS Osteogenesis Imperfecta
*COL1A2*	Collagen type i alpha 2	AD, AR	EDS, type VIIB Osteogenesis Imperfecta; cvEDS
*COL2A1*	Collagen type ii alpha 1	AD AR	OSMED Stickler syndrome
*COL3A1*	Collagen type iii alpha 1	AD	vEDS
*COL5A1*	Collagen type v alpha 1	AD	cEDS
*COL5A2*	Collagen type v alpha 2	AD	cEDS
*COL9A1*	Collagen type ix alpha 1	AD AR	Stickler syndrome
*COL9A2*	Collagen type ix alpha 2	AD AR	Stickler syndrome
*DSE*	Dermatan sulfate epimerase	AR	mcEDS
*EFEMP2*	Egf containing fibulin-like extracellular matrix Protein 2	AR	Cutis laxa
*ELN*	Elastin	AD	Cutis laxa
*FBLN5*	Fibulin 5	AD, AR	Cutis laxa
*FBN1*	Fibrillin 1	AD	Marfan syndrome
*FBN2*	Fibrillin 2	AD	Congenital contractural arachnodactyly
*FKBP14*	Fk506 binding protein 14	AR	EDS with progressive kyphoscoliosis, myopathy, and hearing loss
*FLNA*	Filamin a	XL	EDS with periventricular heterotopia
*LTBP4*	Latent transforming growth factor beta binding Protein 4	AR	Cutis laxa, autosomal recessive
*MAT2A*	Methionine adenosyltransferase ii, alpha	AD	fTAAD
*MED12*	Mediator complex subunit 12	AD	fTAAD, Lujan syndrome
*MFAP5*	Microfibrillar-associated protein 5	AD	fTAAD
*MYH11*	Myosin, heavy chain 11, smooth muscle	AD	fTAAD
*MYLK*	Myosin light chain kinase	AD	fTAAD
*NOTCH1*	Notch, drosophila, homolog of, 1	AD	fTAAD
*PLOD1*	Procollagen-lysine, 2-oxoglutarate 5-dioxygenase	AR	kEDS
*PRDM5*	Pr domain 5	AR	BCS
*PRKG1*	Protein kinase, cgmp-dependent, regulatory, type i	AD	fTAAD
*PYCR1*	Pyrroline-5-carboxylate reductase 1	AR	Cutis laxa, autosomal recessive
*RIN2*	Ras and rab interactor 2	AR	MACS
*SKI*	V-ski avian sarcoma viral oncogene homolog	AD	Shprintzen-Goldberg syndrome
*SLC2A10*	Solute carrier family 2 (facilitated glucose Transporter), member 10	AR	Arterial tortuosity syndrome
*SLC39A13*	Solute carrier family 39 member 13	AR	EDS, Spondylocheirodysplasia type
*SMAD3*	Mothers against decapentaplegic, drosophila, Homolog of, 3	AD	LDS
*SMAD4*	Mothers against decapentaplegic, drosophila, homolog of, 4	AD	JP/HHT
*TGFB2*	Transforming growth factor, beta-2	AD	LDS
*TGFB3*	Transforming growth factor, beta-3	AD	LDS
*TGFBR1*	Transforming growth factor-beta receptor, type i	AD	LDS
*TGFBR2*	Transforming growth factor-beta receptor, type ii	AD	LDS
	Zinc finger protein 469		
*ZNF469*	Transforming growth factor-beta	AR	BCS

**Table 2:** Summary of Loeys–Dietz types.

Type	Gene	Comments
Type 1	*TGFBR1*	Craniofacial involvement more frequent than in Type 2.
Type 2	*TGFBR2*	Displays at least 2 major signs of vascular Ehlers–Danlos syndrome. No craniofacial anomalies.
Type 3	*SMAD3*	Strong predisposition for osteoarthritis.
Type 4	*TGFB2*	Systemic findings less severe & more similar to Marfan syndrome.
Type 5	*TGFB3*	No remarkable arterial tortuosity or evidence of early dissections.

Source: National Institutes of Health (NIH) genetic and rare diseases information, Orphanet Journal of Rare Diseases (OJRD), National Center for Biotechnology Information (NCBI) gene reviews website.

## References

[1] Brown K.A., Pietenpol J.A., Moses H.L. (2007). A tale of two proteins: Differential roles and regulation of Smad2 and Smad3 in TGF-beta signaling. J. Cell. Biochem..

[2] van de Laar I.M., Oldenburg R.A., Pals G., Roos-Hesselink J.W., de Graaf B.M., Verhagen J.M., Hoedemaekers Y.M., Willemsen R., Severijnen L.A., Venselaar H. (2011). Mutations in SMAD3 cause a syndromic form of aortic aneurysms and dissections with early-onset osteoarthritis. Nat. Genet..

[3] van de Laar I.M., van der Linde D., Oei E.H., Bos P.K., Bessems J.H., Bierma-Zeinstra S.M., van Meer B.L., Pals G., Oldenburg R.A., Bekkers J.A. (2012). Phenotypic spectrum of the SMAD3-related aneurysms-osteoarthritis syndrome. J. Med. Genet..

[4] Macklin S., Mohammed A., Jackson J., Hines S.L., Atwal P.S., Caulfield T. (2018). Personalized molecular modeling for pinpointing associations of protein dysfunction and variants associated with hereditary cancer syndromes. Mol. Genet. Genom. Med..

[5] Richter J.E., Zimmermann M.T., Blackburn P.R., Mohammad A.N., Klee E.W., Pollard L.M., Macmurdo C.F., Atwal P.S., Caulfield T.R. (2018). Protein modeling and clinical description of a novel in-frame GLB1 deletion causing GM1 gangliosidosis type II. Mol. Genet. Genom. Med..

[6] von Roemeling C.A., Caulfield T.R., Marlow L., Bok I., Wen J., Miller J.L., Hughes R., Hazlehurst L., Pinkerton A.B., Radisky D.C. (2018). Accelerated bottom-up drug design platform enables the discovery of novel stearoyl-CoA desaturase 1 inhibitors for cancer therapy. Oncotarget.

[7] Cohen I., Coban M., Shahar A., Sankaran B., Hockla A., Lacham S., Caulfield T.R., Radisky E.S., Papo N. (2019). Disulfide engineering of human Kunitz-type serine protease inhibitors enhances proteolytic stability and target affinity toward mesotrypsin. J. Biol. Chem..

[8] Hines S.L., Mohammad A.N., Jackson J., Macklin S., Caulfield T.R. (2019). Integrative data fusion for comprehensive assessment of a novel CHEK2 variant using combined genomics, imaging, and functional-structural assessments via protein informatics. Mol. Omics.

[9] Hines S.L., Richter J.E., Mohammad A.N., Mahim J., Atwal P.S., Caulfield T.R. (2019). Protein informatics combined with multiple data sources enriches the clinical characterization of novel TRPV4 variant causing an intermediate skeletal dysplasia. Mol. Genet. Genom. Med..

[10] Altschul S.F., Madden T.L., Schaffer A.A., Zhang J., Zhang Z., Miller W., Lipman D.J. (1997). Gapped BLAST and PSI-BLAST: A new generation of protein database search programs. Nucleic Acids Res..

[11] Hooft R.W., Sander C., Scharf M., Vriend G. (1996). The PDBFINDER database: A summary of PDB, DSSP and HSSP information with added value. Comput. Appl. Biosci..

[12] Hooft R.W., Vriend G., Sander C., Abola E.E. (1996). Errors in protein structures. Nature.

[13] King R.D., Sternberg M.J. (1996). Identification and application of the concepts important for accurate and reliable protein secondary structure prediction. Protein Sci..

[14] Krieger E., Joo K., Lee J., Lee J., Raman S., Thompson J., Tyka M., Baker D., Karplus K. (2009). Improving physical realism, stereochemistry, and side-chain accuracy in homology modeling: Four approaches that performed well in CASP8. Proteins.

[15] Qiu J., Elber R. (2006). SSALN: An alignment algorithm using structure-dependent substitution matrices and gap penalties learned from structurally aligned protein pairs. Proteins.

[16] Laskowski R.A., MacArthur M.W., Moss D.S., Thornton J.M. (1993). PROCHECK—A Program to Check the Stereochemical Quality of Protein Structures. J. Appl. Crystallogr..

[17] Humphrey W., Dalke A., Schulten K. (1996). VMD: Visual molecular dynamics. J. Mol. Graph..

[18] Caulfield T., Devkota B. (2012). Motion of transfer RNA from the A/T state into the A-site using docking and simulations. Proteins.

[19] Caulfield T., Medina-Franco J.L. (2011). Molecular dynamics simulations of human DNA methyltransferase 3B with selective inhibitor nanaomycin A. J. Struct. Biol..

[20] Caulfield T.R. (2011). Inter-ring rotation of apolipoprotein A-I protein monomers for the double-belt model using biased molecular dynamics. J. Mol. Graph. Model..

[21] Caulfield T.R., Devkota B., Rollins G.C. (2011). Examinations of tRNA Range of Motion Using Simulations of Cryo-EM Microscopy and X-Ray Data. J. Biophys..

[22] Lopez-Vallejo F., Caulfield T., Martinez-Mayorga K., Giulianotti M.A., Nefzi A., Houghten R.A., Medina-Franco J.L. (2011). Integrating virtual screening and combinatorial chemistry for accelerated drug discovery. Comb. Chem. High Throughput Screen..

[23] Reumers J., Schymkowitz J., Ferkinghoff-Borg J., Stricher F., Serrano L., Rousseau F. (2005). SNPeffect: A database mapping molecular phenotypic effects of human non-synonymous coding SNPs. Nucleic Acids Res..

[24] Schymkowitz J.W., Rousseau F., Martins I.C., Ferkinghoff-Borg J., Stricher F., Serrano L. (2005). Prediction of water and metal binding sites and their affinities by using the Fold-X force field. Proc. Natl. Acad. Sci. USA.

[25] Zhang Y.J., Caulfield T., Xu Y.F., Gendron T.F., Hubbard J., Stetler C., Sasaguri H., Whitelaw E.C., Cai S., Lee W.C. (2013). The dual functions of the extreme N-terminus of TDP-43 in regulating its biological activity and inclusion formation. Hum. Mol. Genet..

[26] Abdul-Hay S.O., Lane A.L., Caulfield T.R., Claussin C., Bertrand J., Masson A., Choudhry S., Fauq A.H., Maharvi G.M., Leissring M.A. (2013). Optimization of peptide hydroxamate inhibitors of insulin-degrading enzyme reveals marked substrate-selectivity. J. Med. Chem..

[27] Ando M., Fiesel F.C., Hudec R., Caulfield T.R., Ogaki K., Gorka-Skoczylas P., Koziorowski D., Friedman A., Chen L., Dawson V.L. (2017). The PINK1 p.I368N mutation affects protein stability and ubiquitin kinase activity. Mol. Neurodegener..

[28] Caulfield T.R., Fiesel F.C., Moussaud-Lamodiere E.L., Dourado D.F., Flores S.C., Springer W. (2014). Phosphorylation by PINK1 Releases the UBL Domain and Initializes the Conformational Opening of the E3 Ubiquitin Ligase Parkin. PLoS Comput. Biol..

[29] Caulfield T.R., Fiesel F.C., Springer W. (2015). Activation of the E3 ubiquitin ligase Parkin. Biochem. Soc. Trans..

[30] Fiesel F.C., Ando M., Hudec R., Hill A.R., Castanedes-Casey M., Caulfield T.R., Moussaud-Lamodiere E.L., Stankowski J.N., Bauer P.O., Lorenzo-Betancor O. (2015). (Patho-)physiological relevance of PINK1-dependent ubiquitin phosphorylation. EMBO Rep..

[31] Fiesel F.C., Caulfield T.R., Moussaud-Lamodiere E.L., Ogaki K., Dourado D.F., Flores S.C., Ross O.A., Springer W. (2015). Structural and Functional Impact of Parkinson Disease-Associated Mutations in the E3 Ubiquitin Ligase Parkin. Hum. Mutat..

[32] Puschmann A., Fiesel F.C., Caulfield T.R., Hudec R., Ando M., Truban D., Hou X., Ogaki K., Heckman M.G., James E.D. (2017). Heterozygous PINK1 p.G411S increases risk of Parkinson’s disease via a dominant-negative mechanism. Brain J. Neurol..

[33] Regalado E.S., Guo D.C., Villamizar C., Avidan N., Gilchrist D., McGillivray B., Clarke L., Bernier F., Santos-Cortez R.L., Leal S.M. (2011). Exome sequencing identifies SMAD3 mutations as a cause of familial thoracic aortic aneurysm and dissection with intracranial and other arterial aneurysms. Circ. Res..

[34] Kutty S., Sengupta P.P., Khandheria B.K. (2012). Patent foramen ovale: The known and the to be known. J. Am. Coll. Cardiol..

[35] Sand K.M., Midelfart A., Thomassen L., Melms A., Wilhelm H., Hoff J.M. (2013). Visual impairment in stroke patients—A review. Acta Neurol. Scand. Suppl..

[36] Tan C.K., Tan E.H., Luo B., Huang C.L., Loo J.S., Choong C., Tan N.S. (2013). SMAD3 deficiency promotes inflammatory aortic aneurysms in angiotensin II-infused mice via activation of iNOS. J. Am. Heart Assoc..

[37] Dai X., Shen J., Annam N.P., Jiang H., Levi E., Schworer C.M., Tromp G., Arora A., Higgins M., Wang X.F. (2015). SMAD3 deficiency promotes vessel wall remodeling, collagen fiber reorganization and leukocyte infiltration in an inflammatory abdominal aortic aneurysm mouse model. Sci. Rep..

[38] Qing J., Zhang Y., Derynck R. (2000). Structural and functional characterization of the transforming growth factor-beta-induced Smad3/c-Jun transcriptional cooperativity. J. Biol. Chem..

